# Polynomial-shaped electrodes for adaptive X-ray optics

**DOI:** 10.1107/S160057752600843X

**Published:** 2026-09-18

**Authors:** Kenneth A. Goldberg, Francesco Marzari, Grant D. Cutler

**Affiliations:** a Advanced Light Source Lawrence Berkeley National Laboratory Berkeley CA94720 USA; b Department of Physics University of Trento via Sommarive 14 Trento38123 Italy; c National Institute for Nuclear Physics (INFN) via Sommarive 14 Trento38123 Italy; d Department of Industrial Engineering University of Trento via Sommarive 14 Trento38123 Italy; Tohoku University, Japan

**Keywords:** adaptive optics, X-ray mirrors, lithium niobate, wavefront

## Abstract

For adaptive mirrors on X-ray beamlines and optical systems, we describe a strategy for arranging a small number of tangentially continuous electrodes with varying electrode widths tailored to improve mirror shape control. This approach, with patterned, compound electrodes, is well suited to the emerging class of lithium niobate mirrors that act as monolithic piezo-bimorphs, without the need for additional bonded or grown actuator elements. The proposed electrodes span the length of the mirror’s clear aperture and have width profiles defined by polynomial shapes. Using a limited number of channels derived from Chebyshev polynomials, the actuators provide access to shape controls and correction modes of increasing polynomial order. We derive an analytic approach to optimize control voltages based on Euler–Bernoulli beam theory, and we show how the modeled characteristic response functions can be applied to fit arbitrary surfaces. Comparison with a conventional, linear array of 12 electrodes shows the relative benefit of each approach.

## Abstract 2

A shaped electrode design for lithium niobate adaptive X-ray mirrors connects actuator degrees of freedom to specific polynomial orders, enabling general shape control and the correction of defocus, coma and fourth-order aberrations with a limited number of inputs.

## 1. Introduction

Coherent X-ray science is made possible by visionary investments in ever-brighter, synchrotron and free-electron laser, short-wavelength light sources (Nugent, 2010 ▸; Chapman, 2023 ▸). And, yet, the realization of ‘diffraction-limited’ beamlines and X-ray optical systems to make the science possible has been carried on the shoulders of ultra-high-quality X-ray mirrors and optical elements (Alcock *et al.*, 2025 ▸). Steady, incremental advances in this area allow researchers to optimize the control and use of X-ray beams with increasing certainty. To achieve high reflectivity, atomically smooth X-ray mirrors are illuminated at glancing angles of incidence. The footprint of narrow beams becomes long in one direction; nanometre-to-micrometre-scale surface profiles focus, shape and steer the reflected light.

In pursuit of electronically controlled adaptive X-ray mirrors, Susini’s early work with piezoceramic actuators (Susini *et al.*, 1995 ▸; Susini *et al.*, 1996 ▸), now 30 years ago, mapped a course that many have followed. Through several generations of adaptive mirrors, the technology has made strides in dynamic control (Alcock *et al.*, 2015 ▸; Nistea *et al.*, 2025 ▸), glue-free actuator bonding (Ichii *et al.*, 2019 ▸; Alcock *et al.*, 2026 ▸) and focusing performance (Mimura *et al.*, 2010 ▸). The recent emergence of lithium niobate (LN) as a substrate material well suited for X-ray optics (Inoue *et al.*, 2024 ▸; Inoue *et al.*, 2025 ▸) has captured significant attention. Heat-treated LN behaves as a monolithic bimorph material, simplifying its fabrication and assembly (Nakamura *et al.*, 1987 ▸). Offering fast, linear actuation, free from hysteresis and creep (Shur *et al.*, 2015 ▸), LN overcomes some of the most significant challenges that arise with piezoceramic actuators (Alcock *et al.*, 2013 ▸). Such mirrors avoid the need for glued, bonded or deposited piezoceramic materials and the requirement for interstitial electrodes to deliver opposite polarization to top and bottom layers.

We have recently derived the equations that describe the bending of a two-layer LN adaptive optic (Goldberg *et al.*, 2026 ▸). Our approach followed Timoshenko’s well known study of the bi-metallic thermostat (Timoshenko, 1925 ▸), a canonical system of two bonded material layers having different thermal expansion properties. We utilized Conrad’s approach, adapting Timoshenko’s treatment to the differential strain induced by piezoelectric actuators (Conrad *et al.*, 2008 ▸).

In essence, the local bending curvature achieved with an initially flat substrate is linear with the piezoelectric strain constant and the applied voltage, and it varies inversely with the square of the material thickness. We further demonstrated that shaping the sagittal width of a longitudinally continuous electrode can vary the tangential curvature in a predictable, designed way. Continuous electrodes can simplify shape control and avoid so-called junction effects that occur at the internal boundaries of actuator arrays (Alcock *et al.*, 2013 ▸; Alcock *et al.*, 2015 ▸).

In this work, we show that a limited set of independently controlled, compound electrodes can be designed with polynomial width profiles to achieve mirror surface shaping, to correct aberrations or to achieve arbitrary profiles up to a given polynomial order.

## 2. Mirror substrate properties

Lithium niobate (LiNbO_3_, LN) is a synthetic, crystalline material that has been studied for many decades and used in a wide variety of optical and opto-mechanical applications, including telecommunications, laser Q-switching, acousto-optic devices, and more (Weis & Gaylord, 1985 ▸; Shur, 2017 ▸). Heat treatment near the Curie temperature produces two oppositely poled ferroelectric domains (Nakamura *et al.*, 1987 ▸), an inherently piezoelectric layered structure that behaves like a monolithic piezoelectric bimorph. As an actuator, LN shows no hysteresis or drift, simplifying shape control.

In LN, the piezoelectric response varies strongly with the crystal orientation (Weis & Gaylord, 1985 ▸). Crystals can be cut to maximize the piezoelectric strain coefficients, providing the strongest response to applied voltage. However, the rotated ‘Y-cut’ orientation at 74.3° provides unidirectional expansion most suitable for X-ray adaptive mirror bending (Marzari *et al.*, 2026 ▸). The mirror’s axes can be aligned relative to this crystal orientation for shape control strictly in the tangential direction (with zero sagittal coupling). In this orientation, *d*_21_ is the significant coefficient of the piezoelectric strain tensor, and its value is −6.6453 × 10^−12^ m V^−1^.

## 3. Shaped electrodes

One compelling advantage of LN as an adaptive mirror system is that the electrodes can be deposited as thin metal films and patterned using simple contact photolithography. We consider a geometry in which the layouts of the top and bottom electrodes match.

For a flat, two-layered LN system with a uniform, rectangular cross section and electrodes on the top and bottom faces, we can predict the surface curvature when voltage is applied to the electrodes, as follows. Assume the layer thicknesses are *a*_1_ and *a*_2_, and the total thickness *h* = *a*_1_ + *a*_2_. (The electrode thicknesses are negligible.) A voltage *U* is applied to the top electrode with the bottom electrode grounded; or, equivalently, voltages +*U*/2 and −*U*/2 are applied to the electrodes, respectively. Relative to the full sagittal width, the fractional electrode width β varies as a function of the meridional (tangential direction) position, *Y*. The local curvature κ = 1/ρ, equal to the reciprocal of the local bending radius ρ, varies as (Goldberg *et al.*, 2026 ▸) 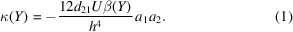
The case of equal layer thicknesses reduces to an expression that we will use in the following analysis,
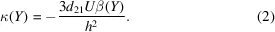
In this simplification, small shape dependencies on the sagittal electrode positions are ignored; however, to avoid twisting forces, we impose a mirror symmetry in the electrodes about the central meridian.

### 3.1. Compound electrodes

Consider a compound set of electrodes made from adjacent, continuous, independent stripes running in the long, tangential direction of the mirror. [This is a notably different arrangement than the segmented linear-array geometries most commonly reported (Inoue *et al.*, 2021 ▸; Inoue *et al.*, 2024 ▸; Nistea *et al.*, 2025 ▸), and discussed in Section 6.] When a single voltage is applied to multiple actuators, the function β(*Y*) becomes the sum of fractional widths from the individual electrodes.

We generalize this approach for *N* adjacent, electrically isolated, independently addressable actuators. The *i*th actuator has a width profile β_*i*_(*Y*) and an applied voltage *u*_*i*_. In equation (2), *U*β(*Y*) becomes 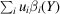, 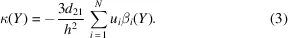
With multiple independent electrodes, applying voltages as +*u*_*i*_/2 and −*u*_*i*_/2 on the complementary, opposing electrodes maintains a symmetric ‘zero’ potential along the central plane, independent of different voltages applied to adjacent electrodes.

### 3.2. Electrode shapes from Chebyshev polynomials

To achieve a variety of potential mirror surface shapes, it is useful to define a set of distinct electrode shapes {*B*_*i*_(*Y*)} and ‘build’ actuated surface profiles from combinations of applied voltages.

**Figure 1:**
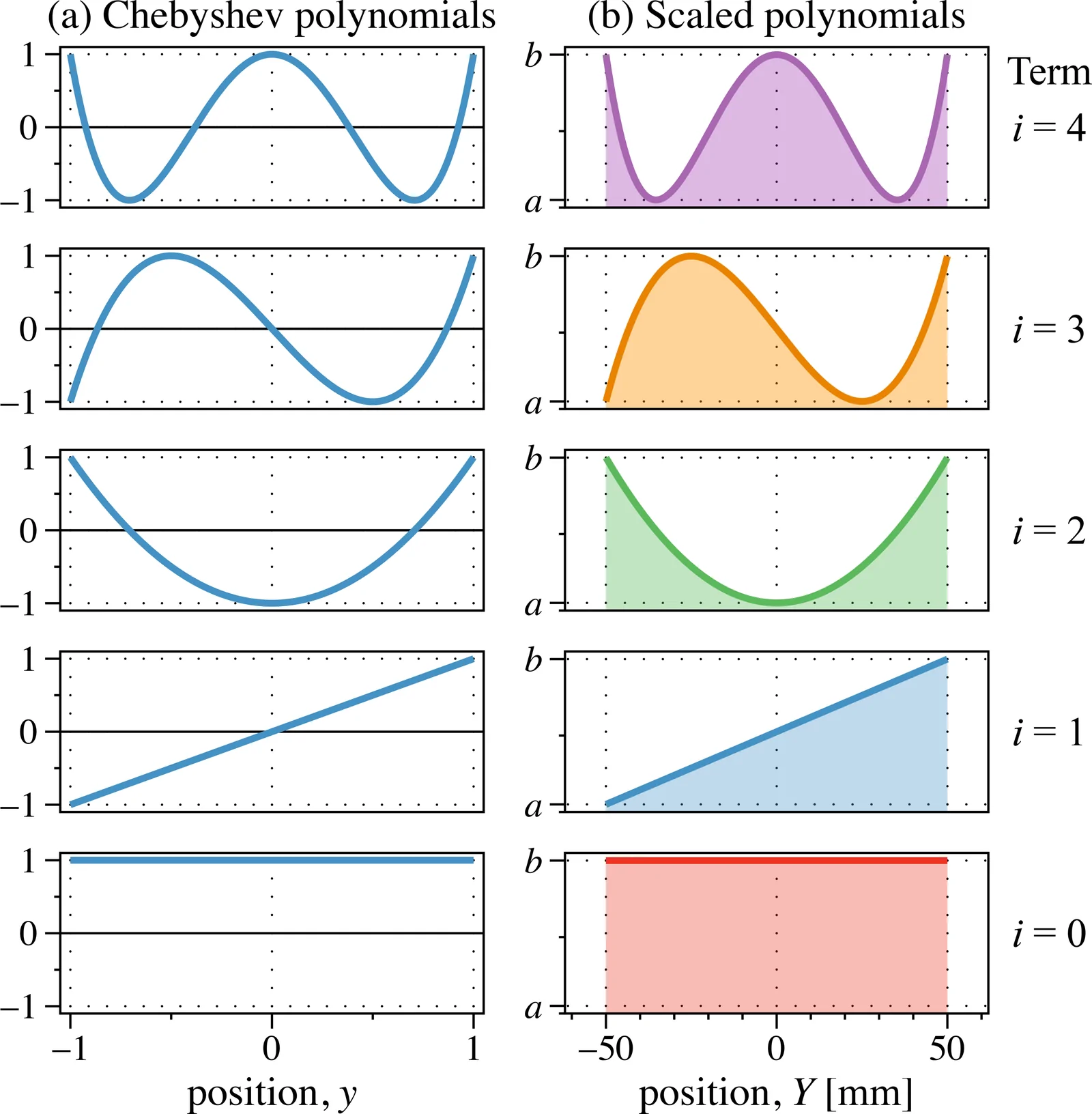
(*a*) The first five Chebyshev polynomials of the first kind, *T*_*i*_(*y*). (*b*) A set of corresponding electrode shapes *B*_*i*_(*Y*) created from scaled and offset Chebyshev polynomials with aperture *l* = 100 mm [equation (4)]. The shapes maintain a minimum width *a* = 0.1 mm in a lane of width *b* = 2.0 mm. The first three terms are used in the electrode design (Fig. 2 ▸) for the demonstration in Section 5.

Various polynomial or trigonometric shapes could be used to form a basis set. Possible types include monomial, Legendre, Chebyshev, Jacobi, Fourier and more. The Chebyshev polynomials of the first kind (Rivlin, 2020 ▸; Hamming, 1986 ▸) are an appropriate set of one-dimensional functions on the domain [−1, 1], with increasing polynomial order. They are conveniently bounded on the range [−1, 1], simplifying scaling in this application. The first five terms are given in Table 1 ▸ and shown in Fig. 1 ▸(*a*).

Physically, an electrode cannot reverse polarity along its length to match functions with positive and negative values. We address this limitation by adding a uniform width to each term, ensuring that they preserve a minimum width, *a*, along their length. In addition, we scale the amplitudes to fit in a defined *lane* of maximum width, *b*.

**Figure 2:**
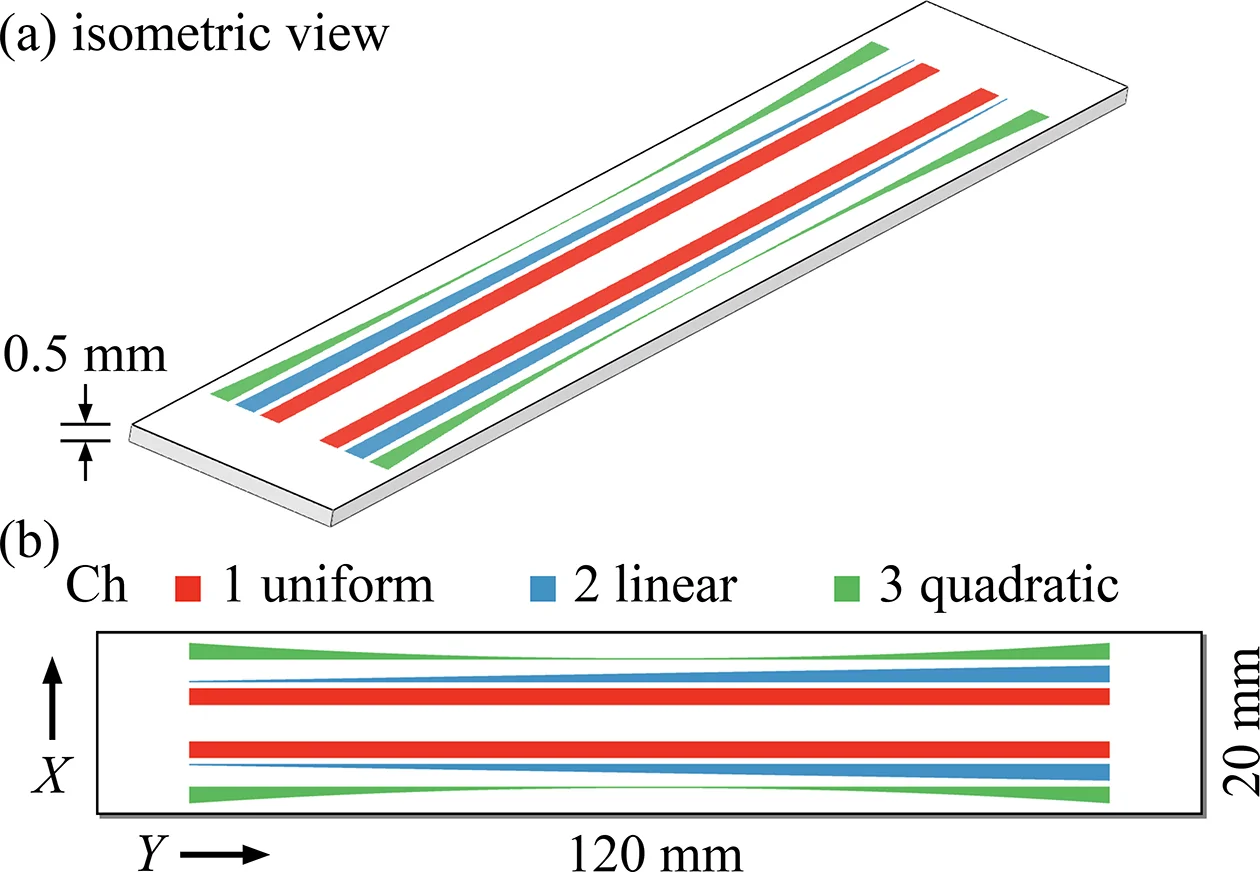
Pattern of six symmetric polynomial-shaped electrodes in three pairs, based on the first three scaled and offset Chebyshev polynomials of the first kind. (*a*) Isometric view. (*b*) Top view. Three pairs of electrodes are labeled in color as channels 1, 2 and 3. A matching electrode pattern is applied to the (unseen) back side. The mirror is 0.5 mm thick.

Working with Chebyshev polynomials, it is helpful to normalize the meridional coordinate, *Y*. With an aperture length *l* (less than the full mirror length *L*), we define the normalized position *y* = 2*Y*/*l*, 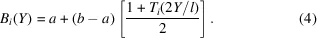
In Fig. 1 ▸(*b*), colored areas represent the electrode shapes that will be scaled and positioned into a symmetric array. Fig. 2 ▸ shows an electrode layout containing six electrodes in three pairs. We maintain a minimum width of *a* = 0.1 mm lanes that are *b* = 2.0 mm wide. On a 20 mm-wide substrate, this lane width fits six channels with 0.5 mm spacing, leaving 4 mm of unpatterened area along the central stripe.

Upon actuation of individual channels, the mirror curvature follows the corresponding polynomial shapes of order *i*, while the mirror shape follows the second antiderivative of order *i* + 2. The maximum electrode width of 12 mm gives a maximum fractional width of β = 0.6. Note that individual lane widths are arbitrary, and there is no requirement for equal widths among the channels.

## 4. Calculating actuation voltages

In this section, we calculate the electrode voltages from a desired surface shape in two ways. First, a strictly analytic approach optimizes the voltages to achieve a curvature profile, starting from equation (3). The second approach uses characteristic response functions for each electrode pair, computed with finite-element analysis (FEA). Voltages are optimized to achieve the target shape, assuming a linear response.

### 4.1. Calculating actuation voltages from the predicted curvature

Given a target shape profile across the clear aperture, or the full mirror length, the first step is to calculate the tangential curvature, κ(*Y*). For X-ray optics illuminated at glancing angles of incidence, the curvature is closely approximated by the second derivative.

With the set of shaped electrodes, optimization with linear algebra determines the optimal applied voltages, {*u*_*i*_}. Vector notation simplifies the expressions. Across *N* points in the domain of *Y*, the target curvature is **κ**, and each electrode shape function is **B**_*i*_. These shapes account for the combined width of the electrodes in pairs, where necessary. Let us define a vector **k** that connects the curvature to the physical mirror thickness and the piezoelectric coupling parameter,
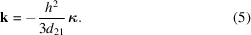
Now equation (3) can be rewritten as 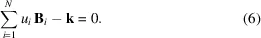
Taking **u** to represent the vector of voltages, we seek a least-squares solution, calculated as a sum over the surface points: ||**Bu** − **k**||^2^. The columns of matrix **B**, indicated by **B**_*i*_, are the electrode widths, {*B*_*i*_(*Y*)}.

The Moore–Penrose matrix inverse (also called the pseudo­inverse) approach solves this set of homogeneous linear equations (**Bu** ≃ **k**) (Moore, 1920 ▸; Penrose, 1955 ▸; Lawson & Hanson, 1995 ▸),
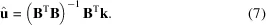
The pseudoinverse matrix, **B**^+^ = (**B**^T^**B**)^−1^**B**^T^, is characteristic of the actuation parameter space and only needs to be calculated once for the system.

Note that a solution based on the curvature requires separate treatment of the constant-offset and the linear-slope as free parameters, since that information is lost when the second derivative is calculated. Arbitrary displacement and tilt can be added mathematically without affecting the curvature; physically, this may require tilt, for example, to maintain beam steering during actuation.

### 4.2. Calculating actuation voltages from the characteristic functions

A well known approach to mirror shape control is to measure the characteristic response from each actuator or degree of freedom. Examples include Hignette’s mirror alignment with *in situ*, pencil beam measurements (Hignette *et al.*, 1997 ▸), McKinney’s application to visible-light mirror metrology (McKinney *et al.*, 2012 ▸) and Merthe’s description for X-ray wavefront-measuring interferometry (Merthe *et al.*, 2012 ▸). The measured differences are also known as *influence functions* (Vannoni *et al.*, 2015 ▸).

The mirror is *L* = 120 mm long, *W* = 20 mm wide and *h* = 0.5 mm thick, matching a commercially available wafer thickness. We modeled the isolated, initially flat mirror’s response to voltages applied to each channel. For this purpose, an *ANSYS* FEA model (Ansys, 2025 ▸) was created to represent the thin, two-layered, LN mirror substrate. The modeling is described in Appendix *A*[App appa]. As voltages are applied to the electrodes, surface shapes are extracted along the central meridian.

**Figure 3:**
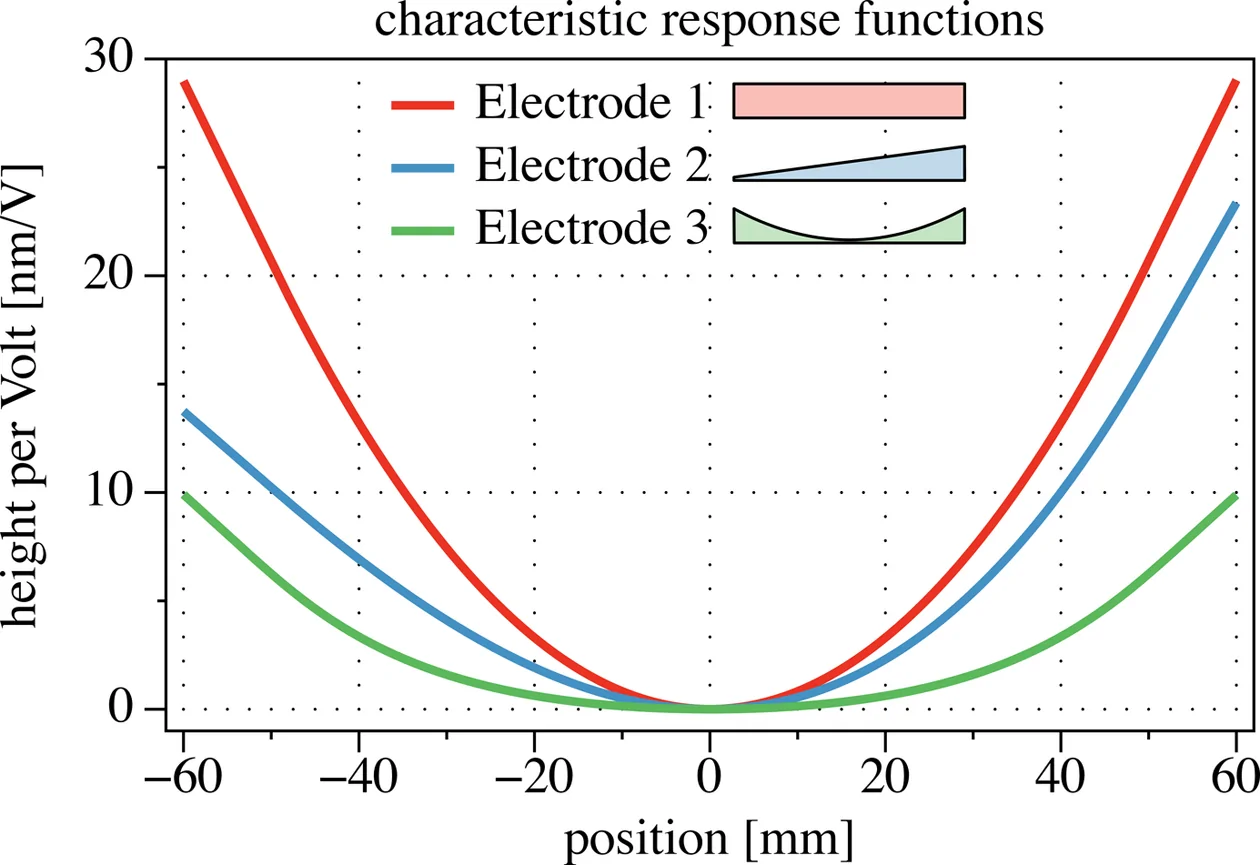
Characteristic response functions {**S**_*i*_}, calculated from an *ANSYS* FEA model. The surface shape profiles are extracted from the central meridian. The curves contain 481 points and represent height change per volt from each of the three electrode pairs. The shapes are plotted with zero central slope and height.

The characteristic functions are shown in Fig. 3 ▸, defined as height change per volt applied. They are plotted with zero central slope and height. As expected, Electrodes 1, 2 and 3 produce shapes that have second-order, third-order and fourth-order polynomial properties, respectively. Electrodes 1 and 3 produce a symmetric response, while the wedge shape of Electrode 2 generates an asymmetric, third-order dependence.

These shapes {**S**_*i*_} are the building blocks of an optimization process that connects applied voltage to surface shape directly, without the intervening curvature calculation of the analytic method. We define **S** as the matrix with columns made from these shapes **S**_*i*_. The matrix requires two additional columns to capture the arbitrary constant-offset and tilt terms: we add a column comprised of 1’s, and a column with a linear ramp from −*L*/2 to *L*/2, or from −*l*/2 to *l*/2, depending on the calculation domain.

For a given mirror shape **a**, solving for the applied voltages using the pseudoinverse is computationally similar to the previous optimization [equation (7)]. We seek electrode voltages 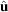 that minimize 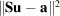:
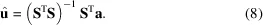
The elements of the vector 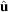 are three voltages, plus the constant-offset and tilt coefficient.

### 4.3. Spatial weighting

The voltage optimizations presented in equations (7) and (8) are for uniform weighting, giving equal importance to all points across the clear aperture. Achieving peak focused beam intensity with a coherently illuminated system requires optimizing the Strehl ratio, as discussed by Goldberg & Yashchuk (2016 ▸). When the beam intensity is non-uniform across the mirror, field-amplitude weighting (*i.e.*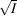) favors the brightest regions of the beam and optimizes the Strehl ratio. Similarly, raytrace analysis of incoherent systems may benefit from simple beam intensity weighting.

Goldberg & La Fleche (2024 ▸) describe the weighted solution. Briefly, with spatial weighting contained in the vector **w**, we create the diagonal square matrix **W** from diag(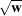). To generate a weighted solution while following the analytic method of Section 4.1, then in equation (7), replace **B** with 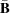 = **W** **B** and **k** with 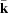 = **W** **k**. If following the method of characteristic functions from Section 4.2, replace **S** with 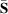 = **W** **S** and **a** with 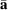 = **W** **a** in equation (8).

## 5. Demonstration from the characteristic functions

**Figure 4:**
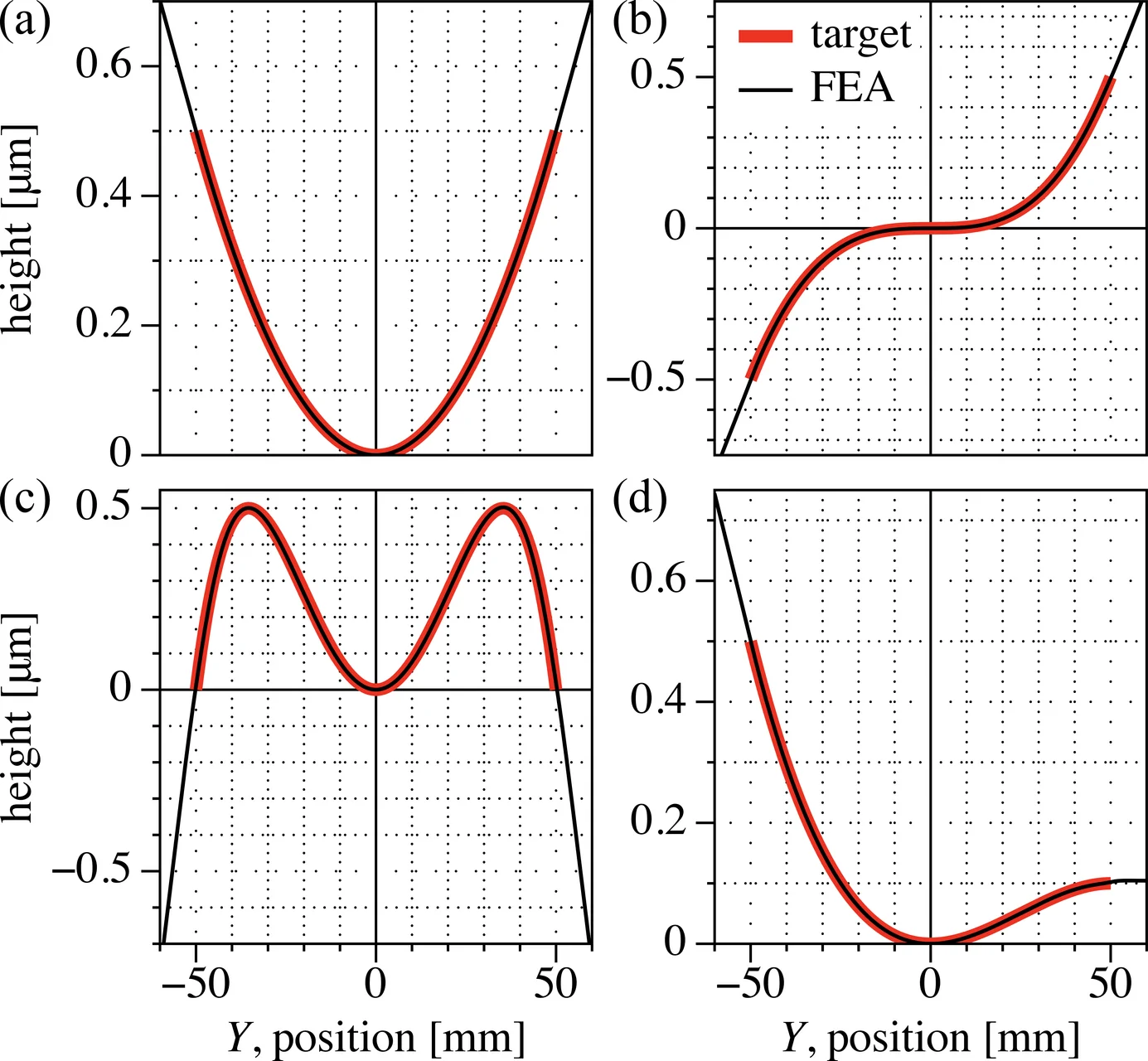
Comparison of target shapes (red lines) and FEA model profiles (black lines). The shapes are defined in Table 2 ▸: (*a*) second order, (*b*) third order, (*c*) fourth order and second order, and (*d*) third order and second order. Electrode voltages used in the model are given in Table 3 ▸. (Note: central heights and slopes have been set to zero.)

Four surface shapes were selected to demonstrate the ability of the three-electrode-pair model to achieve a range of relevant surface profiles. The monomial and binomial shapes are described in Table 2 ▸ and shown as red curves in Fig. 4 ▸. These tests assess the approach described in Section 4.2, using comparisons made across the central 100 mm of the 120 mm-long mirror.

Voltages were calculated from the modeled characteristic functions (Fig. 3 ▸), by applying equation (8) over the central 100 mm region (a domain containing 401 of the total 481 points). These voltages are given in Table 3 ▸: three actuators combine to produce the four modeled shapes. Notably, the wedge-shaped electrode voltages (*u*_2_) take large values only when the shape has a significant asymmetry [cases (*b*) and (*d*)].

The modeled surface profiles are superimposed as black curves on the red target shapes in Fig. 4 ▸. In Table 4 ▸, quantitative comparisons include the r.m.s. magnitudes of the target shapes and the pointwise residual (*i.e.* the difference between the target and the modeled shapes). Because the amplitudes of these test surfaces are arbitrary, it is more significant to consider the ratio of the residual r.m.s. to the shape r.m.s. We find that this ratio is under 0.3% for three of the tests, and is 1.8% for the fourth-order binomial shape, case (*c*).

## 6. Segmented linear electrode array

**Figure 5:**
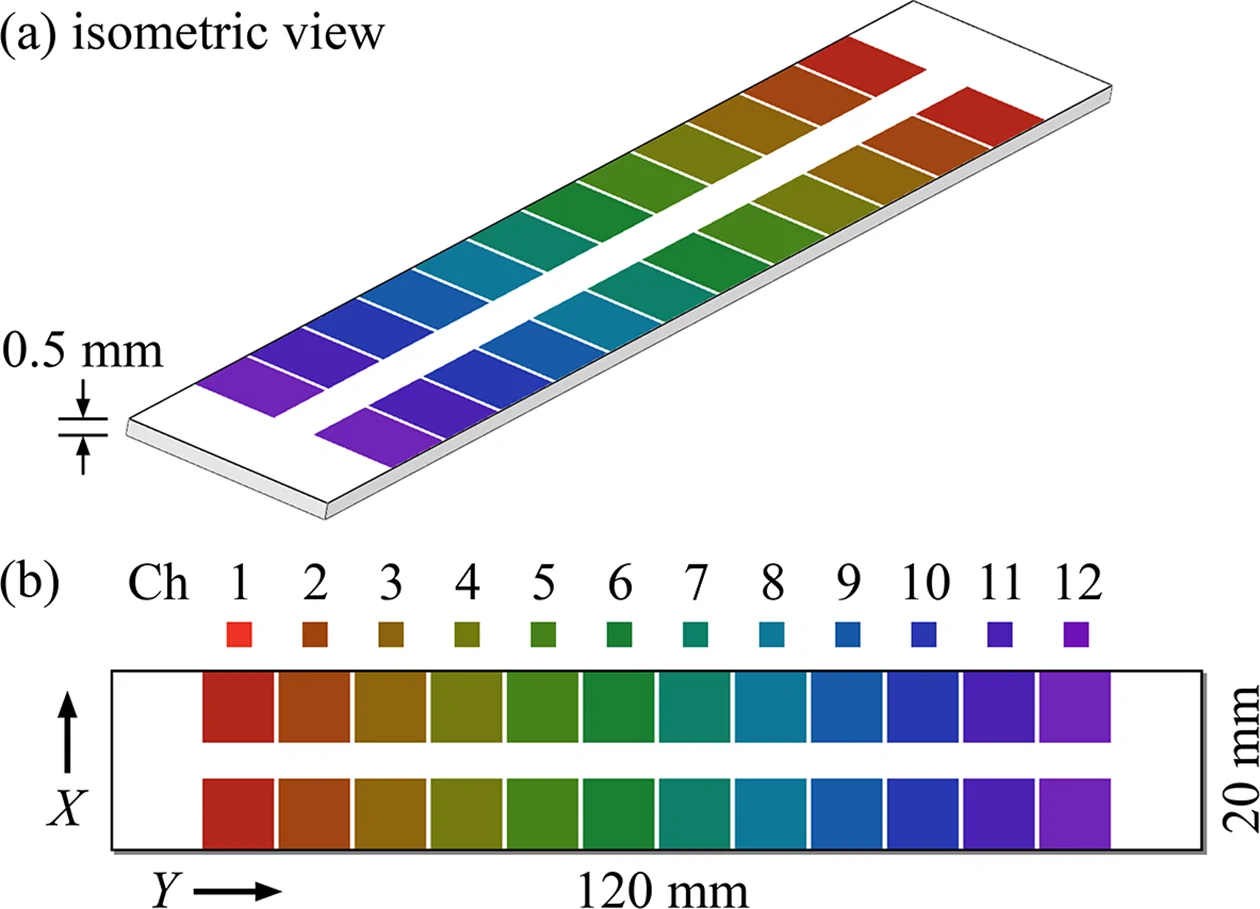
Layout of a 12-channel electrode array, with symmetrically placed electrodes on the front and back sides. The electrodes span the central 100 mm length. The electrodes are labeled as channels 1 through 12. (*a*) Isometric view. (*b*) Top view. A matching electrode pattern is applied to the (unseen) back side. The mirror is 0.5 mm thick.

For comparison, a more conventional segmented linear electrode array design was modeled and tested with the same four target shapes. Fig. 5 ▸ shows a design with 12 individual channels that span 100 mm of the mirror’s length, and a 4 mm, open central region. This electrode layout covers the same region as the polynomial electrodes described above. With a center-to-center distance of 8.375 mm, the individual electrodes are 7.875 mm wide, leaving a 0.5 mm gap between electrodes.

**Figure 6:**
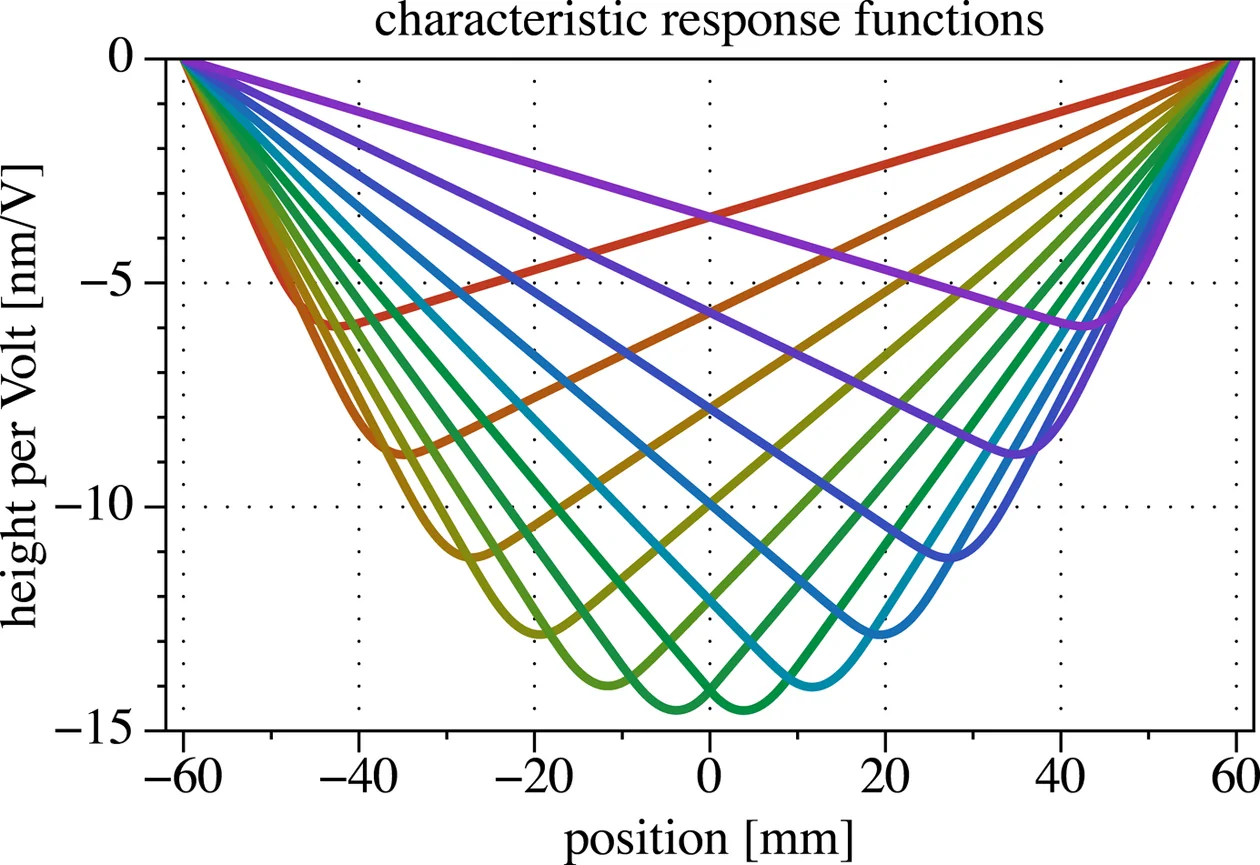
Characteristic response functions of a conventional, 12-channel electrode array, calculated with FEA. The shape profiles are extracted from the central meridian. The curves contain 487 points and represent height change per volt from each channel, individually. Tilt and offset are subtracted from each shape to set the endpoints to zero.

The basis set of characteristic response functions is shown in Fig. 6 ▸, plotted with fixed endpoints to reveal the individual shapes. For each electrode, the characteristic response is roughly triangular, with a narrow region of high curvature at the apex, corresponding to the individual channel positions.

**Figure 7:**
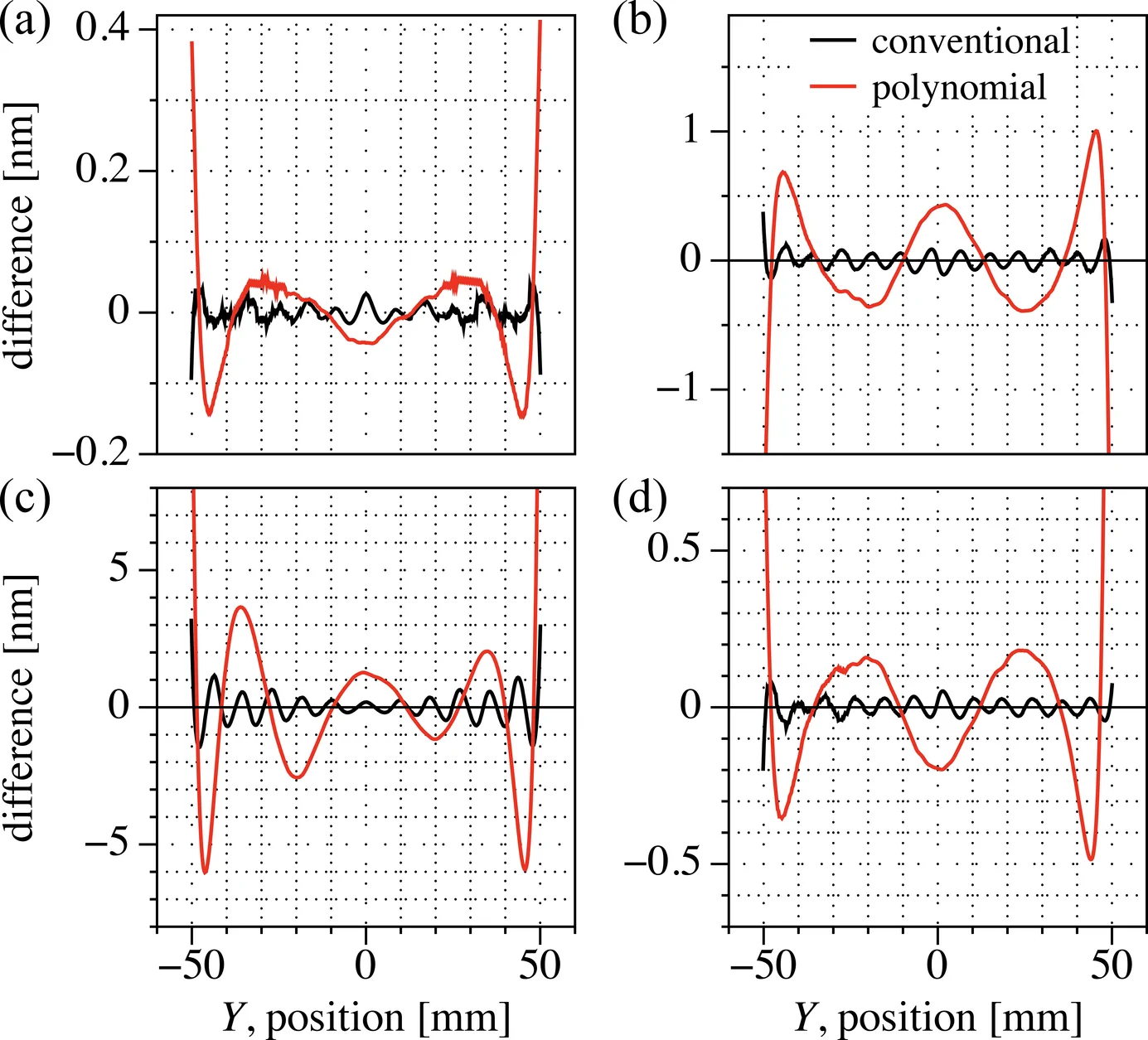
Pointwise residuals between the actuated FEA models and the four target shapes. Red and black lines represent the polynomial electrode model and the linear electrode array model, respectively. Shapes (*a*), (*b*), (*c*) and (*d*) are defined in Table 2 ▸, and the residual r.m.s. values are given in Tables 4 ▸ and 5 ▸. (Note: the tilt and offset terms have been subtracted.)

When actuated to achieve the micrometre-scale target shapes, the FEA-modeled surfaces come within nanometres of their targets over the electrode-containing region—similar to the fitting shown in Fig. 4 ▸. These small residuals are plotted in Fig. 7 ▸ and described quantitatively in Table 5 ▸.

## 7. Discussion

LN offers many advantages for use as an X-ray adaptive mirror substrate, especially in cases where the incident power is low and where a thin substrate of modest length (below 150 mm) can be acceptable. The proposed use of lithographic techniques to create custom, shaped electrodes adds design flexibility that would be challenging or prohibitively difficult to achieve with glued or bonded piezoceramic actuators.

Numerous authors have demonstrated successful, adaptive mirrors with uniformly spaced arrays of segmented actuators that run along the length of the mirror (Signorato *et al.*, 1998 ▸; Mimura *et al.*, 2010 ▸; Alcock *et al.*, 2019 ▸; Shi *et al.*, 2020 ▸; Alcock *et al.*, 2026 ▸). Mirrors with five to 32 actuators, as reported in the literature, are capable of approximating a wide range of surface profiles.

Through analytic and FEA modeling, we have demonstrated a different approach to shape control. Our method leverages the freedom to design electrodes of variable width to create a small number of tangentially continuous, shaped actuators that induce surface profiles of specific polynomial orders. We showed that combinations of these actuators can be programmed to compensate or induce combinations of defocus (second order), coma (third order) and fourth-order wavefront aberrations (and more) in one dimension, using as few as three voltages. The approach can be applied to bending mirrors from an initial flat state, or to providing small compensations to prefigured substrates.

There are trade-offs in selecting the optimal number of actuators in our approach. With more actuators, higher levels of shape control are possible. Yet, with more actuators, the relative sagittal width of each must be reduced and thus the voltage required to produce a given curvature would increase [equation (2)]. In addition, each actuator adds complexity to the assembly, requiring electrical contacts, wires, feedthroughs and control channels from a voltage source.

The polynomial electrodes, with three degrees of freedom, generate fourth-order surface shapes and leave fifth-order and higher residuals. By comparison, the segmented array of 12 electrodes enables smaller residuals, characterized by 24 small ripples. The trade-offs are the relative complexity and the form of the residual shape.

Considering mirrors bent from flat: from equation (2), the minimum curvature radius achievable for a 0.5 mm-thick substrate in this 74.3° rotated Y-cut orientation, with a full-width electrode and 800 V applied is 15.67 m. This cut angle was selected for modeling because applied voltage does not induce sagittal curvature (Marzari *et al.*, 2026 ▸), but cuts with stronger coupling constants are available. In fact, significantly smaller radii have recently been reported (Inoue *et al.*, 2025 ▸).

For shaped electrodes, the achievable curvature varies linearly with the electrode width (Goldberg *et al.*, 2026 ▸). It may not be safe or practical to work with voltages this high. However, Inoue *et al.* have demonstrated applied voltages up to 800 V (Inoue *et al.*, 2025 ▸) with substrates of this same, 0.5 mm thickness. Voltages required for mirror shape or wavefront *correction* could be two or three orders of magnitude smaller when only nanometre-scale actuation is required.

Shape control, in the approach described here, does not overcome the need shared by all X-ray mirrors to begin with surfaces of exceptionally high quality. These can be flat substrates bent or actuated into the optimal shape, or pre­figured mirrors with actuators used for fine control. Depending on the operating wavelength and angle of incidence, nanometre and 10 nrad surface shape and slope quality should be a starting point for advanced performance specifications. To promote high reflectivity and to minimize scattering, mirrors with surface finish quality below 1 nm r.m.s. are required. Achieving these specifications with LN substrates is a relatively new development (Inoue *et al.*, 2024 ▸). Commercially available LN wafers, for example, commonly have warp and bow specifications on the order of 40 µm, far from X-ray mirror figure requirements, despite having high finish quality. The shape of a thin substrate is vulnerable to stress-induced deformation from coatings and processing, and mechanical deformation from mounting.

Regarding fabrication, the error budget for shaped electrode widths depends strongly on the application. In general, relative errors in the electrode width couple into curvature errors in the actuated part (Goldberg *et al.*, 2026 ▸). Modeling reveals that the effect of the actuated bending spreads laterally, by mm, away from the electrode edges. Error tolerance at various spatial length scales is a topic for further investigation. Generally speaking, the magnitude of the shape control errors will scale linearly with the induced deflection. That is, a mirror bent from flat with micrometres of change will be far more sensitive to small imperfections than a mirror whose actuators provide nanometre-scale shape corrections.

While the calculations described here model an ideal mirror system with linear responses, real-world applications must contend with mechanical limitations (supports, mounts, stands *etc*.), finite actuation response times, thermomechanical effects from X-ray power loads and ambient temperature changes, and more. The present work can be a starting point for more sophisticated control systems.

## Figures and Tables

**Table 1:** Chebyshev polynomials of the first kind, *T*_*i*_(*y*), up to fourth order We use the first three terms to design the electrode shapes in the demonstration (Section 5).

*i*	*T*_*i*_(*y*)
0	1
1	*y*
2	2*y*^2^ − 1
3	4*y*^3^ − 3*y*
4	8*y*^4^ − 8*y*^2^ + 1

**Table 2:** Target shapes modeled in Fig. 4 ▸ are defined across the central 100 mm using a normalized position coordinate, *y* = *Y*/50 mm (the leading coefficients are given in micrometres)

Case	Form (µm)
(*a*) 2nd order	0.5 *y*^2^
(*b*) 3rd order	0.5 *y*^3^
(*c*) 4th and 2nd order	2.0 (*y*^2^ − *y*^4^)
(*d*) 3rd and 2nd order	0.1 (3*y*^2^ − 2*y*^3^)

**Table 3:** Electrode voltages (V) used in the four test cases shown in Fig. 4 ▸

Case	*u*_1_	*u*_2_	*u*_3_
(*a*) 2nd order	24.391	−0.109	−0.387
(*b*) 3rd order	−106.498	166.782	−1.421
(*c*) 4th and 2nd order	211.900	−0.275	−698.343
(*d*) 3rd and 2nd order	57.234	−66.778	0.336

**Table 4:** Comparison of the target surface profile shapes to the modeled shapes of the polynomial electrodes across the 100 mm, electrode-containing subdomain ‘Shape r.m.s.’ refers to the target shapes. The ‘residual r.m.s.’ values are based on the residual shape.

	Shape r.m.s. (nm)	Residual r.m.s. (nm)	Ratio (%)
(*a*) 2nd order	149.815	0.074	0.049
(*b*) 3rd order	190.399	0.542	0.285
(*c*) 4th and 2nd order	174.876	3.096	1.771
(*d*) 3rd and 2nd order	117.815	0.259	0.220

**Table 5:** Comparison of the target surface profile shapes to the FEA-modeled shapes of the linear electrode array across the 100 mm, electrode-containing subdomain ‘Shape r.m.s.’ refers to the target shapes. The ‘residual r.m.s.’ values are based on pointwise comparisons.

	Shape r.m.s. (nm)	Residual r.m.s. (nm)	Ratio (%)
(*a*) 2nd order	149.815	0.014	0.009
(*b*) 3rd order	190.399	0.065	0.034
(*c*) 4th and 2nd order	174.876	0.540	0.308
(*d*) 3rd and 2nd order	117.815	0.028	0.023

## Data Availability

Data are available from the authors upon reasonable request.

## Appendix A Finite-element analysis modeling

Coupled field structural-electrostatic elements were used for the analysis. As explained in Section 2, we chose the 74.3° Y-cut orientation for unidirectional bending in the mirror’s long direction.

The model defines the elastic stiffness, relative permittivity and piezoelectric strain coefficients for the two layers of opposite polarization that comprise the monolithic substrate.

The orientation-dependent physical properties of LN were implemented by defining a custom material for both layers. Its elastic stiffness, relative permittivity and piezoelectric strain coefficients are summarized as follows:

Elastic stiffness (GPa):

Relative permittivity:

Piezoelectric strain coefficients (pm V^−1^):

While the elastic stiffness and relative permittivity remain identical for both substrate layers, the piezoelectric strain coefficients of the inversion layer are of opposite sign relative to the original values.

The electromechanical response of LN follows the linear theory of piezoelectricity. As mentioned above, we modeled the electrodes as boundary conditions with a voltage +*U*/2 applied to the top electrode and −*U*/2 to the bottom. The mirror is free to deform with no external structural constraints. Structural symmetry boundary conditions were applied to the planes passing through the mirror center, oriented parallel to the tangential and sagittal directions, and normal to the mirror surface.

Examination of the full, modeled surfaces during actuation shows that ‘edge effects’, manifesting as curvature deviation, extend approximately 10 mm from the ends of the mirror. Smaller sagittal shape variations also occur with dependence on the lateral placement of the symmetric actuator pairs. These effects become part of the shape optimization calculations when the characteristic-function approach is applied (Section 4.2).
